# Transport of phenoxyacetic acid herbicides by PIN-FORMED auxin transporters

**DOI:** 10.1038/s41477-025-01984-0

**Published:** 2025-04-22

**Authors:** Lukas Schulz, Kien Lam Ung, Lorena Zuzic, Sarah Koutnik-Abele, Birgit Schiøtt, David L. Stokes, Bjørn Panyella Pedersen, Ulrich Z. Hammes

**Affiliations:** 1 Plant Systems Biology, School of Life Sciences Weihenstephan, Technical University of Munich, Freising, Germany; 2 Department of Molecular Biology and Genetics, Aarhus University, Aarhus, Denmark; 3 Department of Chemistry, Aarhus University, Aarhus, Denmark; 4 Institute for Advanced Study, Technical University of Munich, Garching, Germany; 5 Department of Biochemistry and Molecular Pharmacology, New York University School of Medicine, New York, NY USA; 6 Present Address: Institut de Pharmacologie et de Biologie Structurale (IPBS), Université de Toulouse, CNRS, UPS, Toulouse, France

**Keywords:** Auxin, Cryoelectron microscopy

## Abstract

Auxins are a group of phytohormones that control plant growth and development. Their crucial role in plant physiology has inspired development of potent synthetic auxins that can be used as herbicides. Phenoxyacetic acid derivatives are a widely used group of auxin herbicides in agriculture and research. Despite their prevalence, the identity of the transporters required for distribution of these herbicides in plants is both poorly understood and the subject of controversial debate. Here we show that PIN-FORMED auxin transporters transport a range of phenoxyacetic acid herbicides across the membrane. We go on to characterize the molecular determinants of substrate specificity using a variety of different substrates as well as protein mutagenesis to probe the binding site. Finally, we present cryogenic electron microscopy structures of *Arabidopsis thaliana* PIN8 bound to either 2,4-dichlorophenoxyacetic acid or 4-chlorophenoxyacetic acid. These structures represent five key states from the transport cycle, allowing us to describe conformational changes associated with the transport cycle. Overall, our results reveal that phenoxyacetic acid herbicides use the same export machinery as endogenous auxins and exemplify how transporter binding sites undergo transformations that dictate substrate specificity. These results provide a foundation for future development of novel synthetic auxins and for precision breeding of herbicide-resistant crop plants.

## Abstract (web summary)

PIN transporters are key players in distributing phenoxyacetic acid herbicides. Mutagenesis and cryo-EM structures elucidate substrate specificity and transport mechanisms, paving the way for improved synthetic auxin development and herbicide-resistant crops.

## Main

Auxins are a group of plant hormones that regulate development and growth responses in plants. These hormones are distributed through a process known as polar auxin transport, which underlies fundamental environmental responses such as gravitropic and phototropic growth, root development and lateral root initiation, as well as organ initiation and differentiation^1–3^. PIN-FORMED (PIN) auxin transporters are key for providing polarity to auxin transport by mediating auxin export from the cytosol to the apoplast^4,5^. The principal endogenous auxin is indole-3-acetic acid (IAA, acid dissociation constant p*K*_a_ = 4.7), and a variety of synthetic auxins are used as herbicides, together accounting for ~20% of all herbicide-treated farmland worldwide^6^. In general, the synthetic auxins elicit typical auxin responses, but with stronger effects that are due in part to longer half-lives in the cytosol^6–9^. A major subgroup of synthetic auxins comprises phenoxyacetic acids such as 4-chlorophenoxyacetic acid (4-CPA, p*K*_a_ = 3.56) and the widely used herbicide 2,4-dichlorophenoxyacetic acid (2,4-D, p*K*_a_ = 2.73). Auxin herbicides show selective action and are mainly used to control dicot weeds in monocot cereal crops^8^. Despite their high agricultural and ecological impact, it remains unclear how these compounds are distributed across plant tissues. It remains controversial whether PINs are the responsible exporters, and this ambiguity makes it impossible to understand how the observed chemical diversity of synthetic auxins is tolerated^10^.

Recently, structures of *Arabidopsis thaliana* PIN8, PIN1 and PIN3 have been determined, revealing homodimers in which each monomer harbours ten transmembrane helices (M1–M10) with an inverted repeat that produces two 5-helix bundles^10–13^. The first two helices from each repeat (M1–M2 and M6–M7) form a scaffold domain that mediates dimerization, and a long cytoplasmic loop between M5 and M6 that connects the two repeats. A transport domain is composed of M3–M5 and M8–M10, also related by the inverted symmetry, and a substrate binding chamber is centred on the crossover of M4 and M9, which form interrupted α-helices^11,12^. PIN8 produced structures in both inward and outward conformations, thus showing that PINs operate by an elevator mechanism where the substrate-loaded transporter domain is translated 5 Å across the membrane relative to the scaffold domain^12,14^. In the inward open conformation, the binding chamber connects to a larger vestibule leading to the cytosol, whereas in the outward open conformation the binding chamber connects directly to the extracellular space^11,12^. All existing structures show symmetric dimers in which monomers adopt identical conformations^11–13,15^.

In this Article, we address phenoxyacetic acid herbicide transport and broader implications for substrate recognition in PINs. We show that PINs can bind and transport these synthetic auxins in vitro and that these substrates are distributed in planta in a PIN-dependent fashion. In addition, we characterize binding and transport properties of a variety of related natural and synthetic auxins in vitro, and we use single-particle cryogenic electron microscopy (cryo-EM) to solve structures of PIN8 in complex with 2,4-D or 4-CPA to confirm their role as PIN substrates and to clarify how the chemical diversity of synthetic auxins can be tolerated in the binding site. In the case of 4-CPA, a single sample produced five different structures representing key binding intermediates from the transport cycle and indicating that PIN monomers operate asynchronously. Together with biochemical characterization the structures pinpoint key elements of PIN proteins and auxin substrates that control affinity and specificity. This information may be useful in engineering herbicide-resistant crops as well as novel environmentally safer herbicides for sustainable agriculture.

## Results

**Fig. 1:**
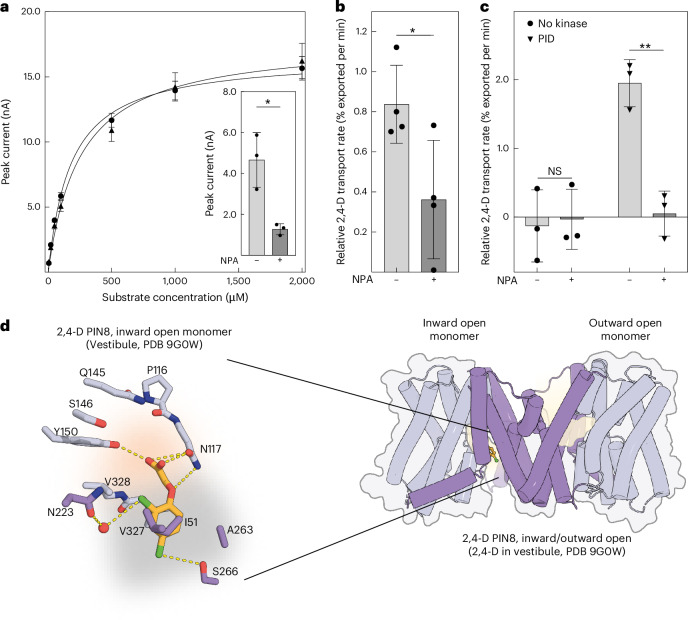
2,4-D is a PIN substrate. **a**, Peak current response as a function of the concentrations of IAA (filled triangles) and 2,4-D (filled circles) determined by SSM electrophysiology on PIN8 proteoliposomes. A Michaelis–Menten model is fit to describe kinetics (${r}_{\rm{IAA}}^{2}$ = 0.9579; ${\rm{EC}}_{50}^{\rm{IAA}}$ = 254 ± 6 μM; ${I}_{\max }^{\rm{IAA}}$ = 18 ± 3 nA versus ${r}_{\mathrm{2,4}{\mbox{-}}{\rm{D}}}^{2}$ = 0.9802; ${\rm{EC}}_{50}^{\mathrm{2,4}{\mbox{-}}{\rm{D}}}$ = 179 ± 10 μM; ${I}_{\max }^{\mathrm{2,4}{\mbox{-}}{\rm{D}}}$ = 17 ± 2 nA; data points represent mean ± s.e.m.; *n* = 4 individual sensors). Inset: peak current response elicited by 100 μM 2,4-D in the absence or presence of 20 µM NPA. Data are mean ± s.e.m. of *n* = 3 individual sensors. Groups were compared by a two-sided unpaired Student’s *t*-test. *P = 0.0126. **b**, Relative transport rates of [3H]-2,4-D export by PIN8 in the presence or absence of 10 μM NPA as indicated from oocytes calculated from four independent time course experiments. Data points are individual experiments, and bars represent mean ± s.d. Groups were compared by a two-sided unpaired Student’s *t*-test. *P = 0.0361; *n* = 4 individual experiments. **c**, Relative transport rates of [3H]-2,4-D export by PIN3 with or without the activating PID kinase in the presence or absence of 10 μM NPA as indicated from oocytes calculated from three independent time course experiments. Data points are individual experiments, and bars represent mean ± s.d. Groups were compared by a two-sided unpaired Student’s *t*-test. NS, not significant *P* = 0.6411; **P = 0.0004; *n* = 3 individual experiments. **d**, Structure of asymmetric dimeric PIN8 with 2,4-D bound to the inward open monomer in a prebinding position in the vestibule (right panel). The transporter domain is highlighted in light violet and the scaffold domain in violet, and the ligand is coloured orange. Close-up view of 2,4-D and the residues from the scaffold domain (violet) and transporter domain (light violet) interacting with the substrate. The inward-open pocket is divided into the binding chamber (highlight in light orange) and a vestibule (highlight in grey). Red spheres represent water molecules. Figure created with PyMOL v5.0.4, Molecular Graphics System (Schrödinger, LLC). Source data

**Extended Data Fig. 1:**
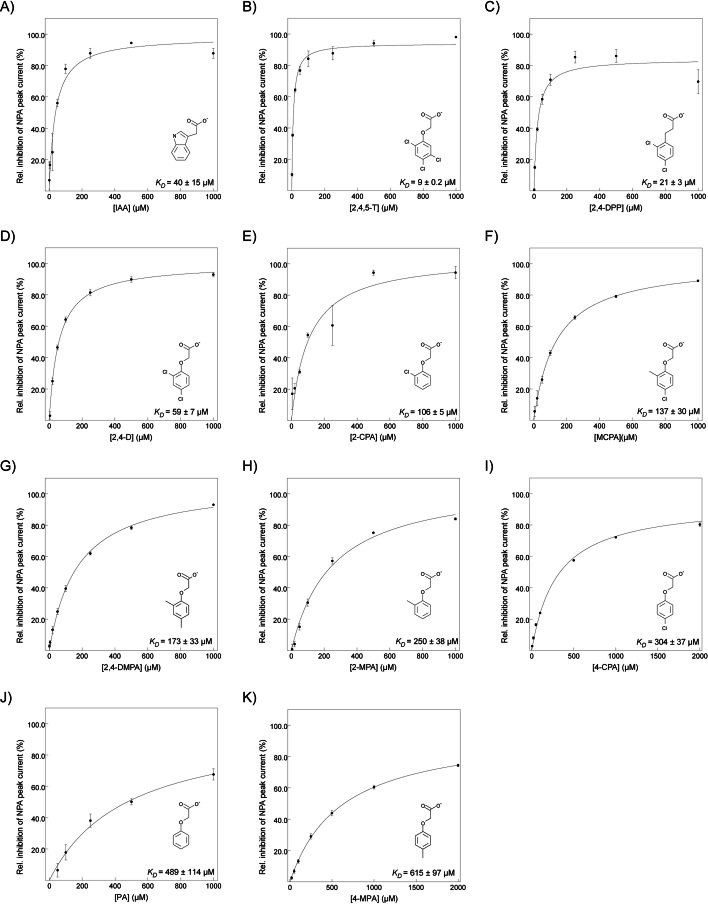
Full *K*_*D*_ measurements of substrates. *K*_*D*_ of substrate binding by PIN8 determined by SSM electrophysiology. Relative inhibition of Peak current response elicited by 100 µM NPA by [substrate] indicated. A Michaelis–Menten model is fit to describe kinetics; data points represent mean ± s.e.m. **a**) IAA (${r}_{{IAA}}^{2}$ = 0.91, ${K}_{D}^{{IAA}}$ 40 ± 15 μM; n = 3 individual sensors) **b**) 2,4,5-T (${r}_{2,4,5-T}^{2}$ = 0.98, ${K}_{D}^{2,4,5-T}$ 9 ± 0.2 μM; n = 3 individual sensors) **c**) 2,4-DPP (${r}_{\mathrm{2,4}-{DPP}}^{2}$ = 0.93, ${K}_{D}^{\mathrm{2,4}-{DPP}}$ 21 ± 3 μM; n = 3 individual sensors). **d**) 2,4-D (${r}_{\mathrm{2,4}D}^{2}$ = 0.99, ${K}_{D}^{\mathrm{2,4}D}$ 59 ± 7 μM; n = 3 individual sensors). **e**) 2-CPA (${r}_{2-{CPA}}^{2}$ = 0.89, ${K}_{D}^{2-{CPA}}$ 106 ± 5 μM; n = 3 individual sensors) **f**) MCPA (${r}_{{MCPA}}^{2}$ = 0.99, ${K}_{D}^{{MCPA}}$ 137 ± 30 μM; n = 3 individual sensors) **g**) 2,4-DMPA (${r}_{\mathrm{2,4}-{DMPA}}^{2}$ = 0.99, ${K}_{D}^{\mathrm{2,4}-{DMPA}}$ 174 ± 33 μM; n = 3 individual sensors) **h**) 2-MPA (${r}_{2-{MPA}}^{2}$ = 0.99, ${K}_{D}^{2-{MPA}}$ 250 ± 38 μM; n = 3 individual sensors) **i**) 4-CPA (${r}_{4-{CPA}}^{2}$ = 0.99, ${K}_{D}^{4-{CPA}}$ 304 ± 37 μM; n = 3 individual sensors) **j**) PA (${r}_{{PA}}^{2}$ = 0.95, ${K}_{D}^{{PA}}$ 489 ± 114 μM; n = 3 individual sensors) **k**) 4-MPA (${r}_{4-{MPA}}^{2}$ = 0.99, ${K}_{D}^{4-{MPA}}$ 21 ± 3 μM; n = 3 individual sensors). Source data

**Extended Data Fig. 2:**
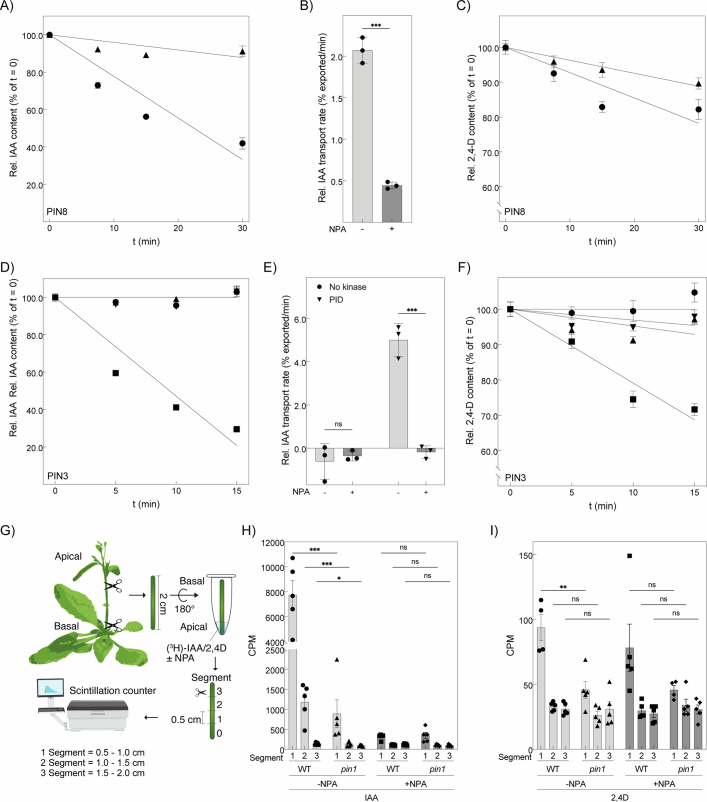
PIN mediated IAA and 2,4-D export from Xenopus oocytes and basipetal auxin transport in inflorescence Arabidopsis stems. **a**) Time course of [3H]-IAA export by PIN8 from oocytes. Data of a typical experiment. Relative IAA content of oocytes expressing PIN8 in the presence (▲) or absence (•) of 10 μM NPA internally determined at the time indicated after substrate injection. Initial internal IAA concentration was 1 μM. n = 10 oocytes at each time point. Data points are mean ± SE. **b**) Relative transport rates of [3H]-IAA export by PIN8 in presence (dark grey) or absence (light grey) of 10 μM NPA as indicted from oocytes calculated from three independent time course experiments. Data points are individual experiments and bars represent mean ± SD. Groups were compared by a two-sided unpaired Student’s t-test; *** P < 0.0001. **c**) Time course of [3H]-2,4-D export by PIN8 from oocytes. Data of a typical experiment. Relative IAA content of oocytes expressing PIN8 in the presence (■) or absence (•) of 10 μM NPA internally determined at the time indicated after substrate injection. Initial internal 2,4-D concentration was 1 μM. n = 10 oocytes at each time point. Data points are mean ± SE. **d**) Time course of [3H]-IAA export by PIN3 from oocytes. Data of a typical experiment. Relative IAA content of oocytes expressing PIN3 without kinase in the presence (■) or absence (•) of 10 μM NPA internally and PID in the presence (▼) or absence (▲) of 10 μM NPA internally determined at the time indicated after substrate injection. Initial internal IAA concentration was 1 μM. n = 10 oocytes at each time point. Data points are mean ± SE. **e**) Relative transport rates of [3H]-IAA export by PIN3 with or without PID in presence (dark grey) or absence (light grey) of 10 μM NPA as indicated from oocytes calculated from three independent time course experiments. Data points are individual experiments and bars represent mean ± SD. Groups were compared by a two-sided unpaired Student’s t-test. n.s. not significant (P =0.99), *** P < 0.0001. **f**) Time course of [3H]-2,4-D export by PIN3 from oocytes. Data of a typical experiment. Relative 2,4-D content of oocytes expressing PIN3 without kinase in the presence (■) or absence (•) of 10 μM NPA internally and PID in the presence (▼) or absence (▲) of 10 μM NPA internally determined at the time indicated after substrate injection. Initial internal 2,4-D concentration was 1 μM. n = 10 oocytes at each time point. Data points are mean ± SE. **g**) Illustration of the stem transport assay measured in inflorescence stems of 5-week-old Arabidopsis plants. 2 cm stem sections were cut above the rosette of 5-week-old plants and placed in an inverted orientation into auxin transport buffer containing [3H]-IAA or [3H]-2,4-D as a substrate in the absence and presence of NPA. After two hours, 5 mm segments were dissected, and the substrate was quantified using a liquid scintillation counter. Segment 1 represents 0.5 to 1 cm, Segment 2 represents 1 to 1.5 cm and Segment 3 represents 1.5 to 2 cm of the stem, from apical to basal position. **h**) [3H]-IAA content in segments of Col-0 wildtype and *pin1* mutant plants in the absence and presence of NPA as indicated. We used n = 5 individual inflorescences for segment analyses. Data points are segments of individual plant stems and bars represent mean ± SD. Groups were compared by a two-sided unpaired Student’s t-test. For individual p-values see Supplementary Table 2. **i**) [3H]-2,4-D content in segments of Col-0 wildtype and *pin1* mutant plants in the absence and presence of NPA as indicated. We used n = 5 segments of individual inflorescences for Col-0 segments 2 and 3 without NPA, Col-0 segments 1, 2 and 3 with NPA, *pin1* segments 1, 2 and 3 without NPA and *pin1* segments 2 and 3 with NPA and n = 4 stem segments of individual inflorescences for Col-0 segment 1 without NPA and *pin1* segment 1 with NPA. Data points are segments of individual plant stems and bars represent mean ± SD. Groups were compared by a two-sided unpaired Student’s t-test. For individual p-values see Supplementary Table 2. Source data

Trans-cellular transport and distribution of 2,4-D in planta has been shown to be active, polar and sensitive to IAA export inhibitors but much slower than IAA transport^16–18^. These results suggested that 2,4-D is transported by PIN proteins. By contrast, one influential study suggested that the low rate of 2,4-D export implies passive diffusion across the membrane rather than a protein-mediated transport process^19^. To investigate this discrepancy and understand the basis for auxin herbicide transport, we used solid supported membrane (SSM) electrophysiology to characterize 2,4-D binding and transport by purified *A. thaliana* PIN8 reconstituted into proteoliposomes (Fig. 1a). PIN8 is closely related to PIN3, and the results obtained for PIN8 align well with those obtained for PIN1 and PIN3, making PIN8 a good proxy to address questions regarding transport mechanism and substrate binding of the PIN family^11–13,15^. We find that the peak current responses elicited by 2,4-D and IAA are similar (half maximal effective concentration ${\rm{EC}}_{50}^{\rm{IAA}}$ = 250 ± 10 µM; maximal current ${I}_{\max }^{\rm{IAA}}$ = 18 ± 3 nA versus ${\rm{EC}}_{50}^{2,4{\mbox{-}}\mathrm{D}}$ = 180 ± 10 µM; ${I}_{\max }^{2,4{\mbox{-}}\mathrm{D}}$ = 17 ± 2 nA) (Fig. 1a). The current response to 2,4-D, like that of IAA, is inhibited by the PIN-inhibitor 1-*N*-naphthylphthalamic acid (NPA), suggesting competitive binding (Fig. 1a inset). Based on this inhibition, we determine the 2,4-D binding affinity (${K}_{\rm{D}}^{2,4{\mbox{-}}\mathrm{D}}$) to be 59 ± 7 µM, which is similar to the previously described IAA binding affinity (${K}_{\rm{D}}^{\rm{IAA}}$) of 40 ± 15 µM (Extended Data Fig. 1a,d and ref. ^12^). These in vitro PIN8 results are corroborated by *Xenopus* oocyte efflux assays using both PIN8 and PIN3 (Fig. 1b,c). These assays show that PIN8 mediates NPA-sensitive 2,4-D export from oocytes with a transport rate of about half of that determined for IAA (Fig. 1b and Extended Data Fig. 2a–c). To show that 2,4-D export is not unique to PIN8, we tested *A. thaliana* PIN3 with and without the activating PINOID (PID) kinase^20^. As expected, PIN3 is activated by kinase, and, as with PIN8, the NPA-sensitive, 2,4-D export rate by PIN3 is about half of the IAA transport rate (Fig. 1c and Extended Data Fig. 2d–f). To test directional transport in plants, we used an *A. thaliana* stem segment assay and compared 2,4-D and IAA transport in stem segments of wild type and *pin1* mutants as PIN1 is the transporter responsible for auxin transport in this organ (Extended Data Fig. 2g–i)^20^. This assay shows that although 2,4-D transport is about 20-fold lower than IAA transport in wild-type plants, it is completely abolished in *pin1* mutants, demonstrating that 2,4-D transport in planta is in fact PIN-mediated.

**Extended Data Fig. 3:**
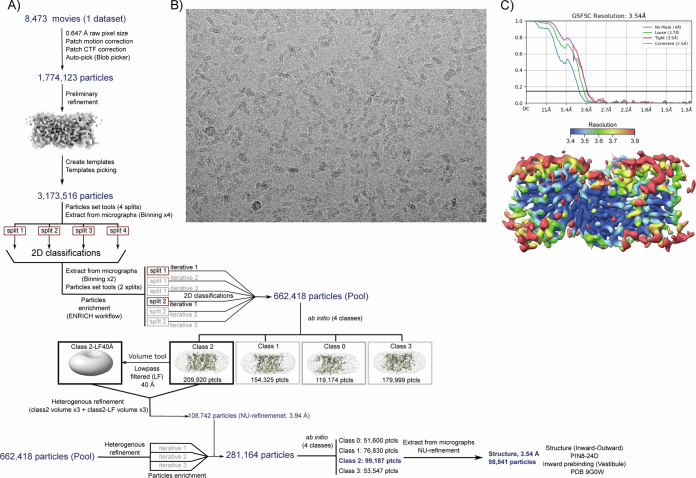
Image processing and reconstruction for 2,4-D bound PIN8. **a**) Workflow of image processing and 3D reconstruction in cryoSPARC. **b**) motion corrected micrograph from Titan Krios microscope using a K3 detector. **c**) sharpened density map from the final non-linear refinement coloured by local resolution. Corrected curve of the global Fourier shell correlation (FSC) indicates 3.54 Å based on the 0.143 gold-standard criterion. Figure created with Chimera v1.15 (ref. ^28^).

**Extended Data Fig. 4:**
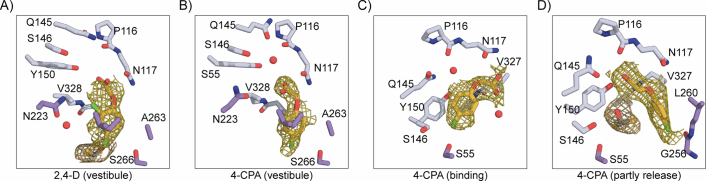
Structures of PIN8 with bound 2,4-D or 4-CPA. Close-up view of 2,4-D and 4-CPA map density and the residues interacting with the ligand. **a**) 2,4-D in the prebinding state in vestibule of the inward monomer **b**) 4-CPA in the prebinding state in vestibule of the inward monomer **c**) 4-CPA in binding state of the outward monomer **d**) 4-CPA in partly release state of the outward monomer. Figure created with PyMOL v5.0.4, Molecular Graphics System (Schrödinger, LLC) .

To supplement the transport data, we use cryo-EM to determine the structure of PIN8 bound to 2,4-D at 3.5 Å resolution (Fig. 1d, Extended Data Figs. 3 and 4a and Supplementary Table 1). It is worth noting that the homo-dimer structure is asymmetric where one monomer adopts an inward facing (IF) conformation and the other an outward facing (OF) conformation. The monomer in the inward conformation shows 2,4-D bound within the vestibule seen in previous structures, with a pose that suggests a prebinding position. In particular, the carboxyl group of 2,4-D interacts with Asn117, and interrupted helices associated with the crossover (M4 and M9) are positioned such that their dipoles neutralize the negative charge. By contrast, the monomer in the outward facing conformation has weak density in the binding pocket that cannot be confidently modelled, but that appears consistent with 2,4-D release (Fig. 1d).

**Fig. 2:**
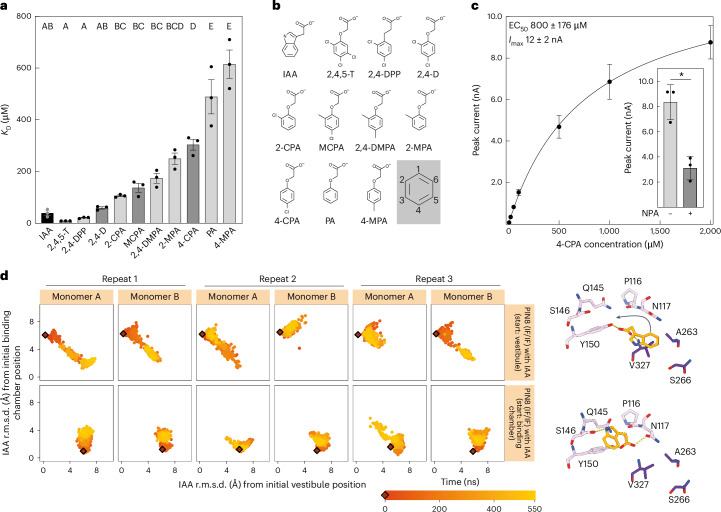
Substrate determinants and 4-CPA binding affinity. **a**, *K*_D_ of substrate binding by PIN8 as determined by SSM electrophysiology. Dark grey represents substrates with herbicidal activity. PA, phenoxyacetic acid; 2-CPA, 2-chlorophenoxyacetic acid; 2-MPA, 2-methylphenoxyacetic acid; 2,4-DPP, 3-(2,4-dichlorophenyl)-propionic acid; 2,4-DMPA, 2-(2,4-dimethylphenoxy)acetic acid; 4-MPA, 4-methylphenoxyacetic acid. Data points are individual sensors, and bars represent mean ± s.e.m. Data points derived from the full measurements (Extended Data Fig. 1). Letters indicate significant differences between groups determined by a one-way ANOVA followed by a Turkey’s multiple comparisons test (*P* < 0.05). For individual *P* values, see Supplementary Table 3. **b**, Chemical structures of the substrates tested are shown at pH 7.4. The grey shaded insert shows the positions in the ring system. **c**, Peak current response as a function of the concentration of 4-CPA determined by SSM electrophysiology on PIN8 proteoliposomes. A Michaelis–Menten model is fit to describe kinetics (${r}_{4{\mbox{-}}\rm{CPA}}^{2}$ = 0.98; ${\rm{EC}}_{50}^{4{\mbox{-}}{\rm{CPA}}}$ = 800 ± 200 μM; ${I}_{\max }^{4{\mbox{-}}\rm{CPA}}$ = 12 ± 2 nA; data points represent mean ± s.e.m.; *n* = 3 individual sensors). Inset: peak current response elicited by 2 mM 4-CPA in the absence or presence of 20 µM NPA. Data are mean ± s.e.m. of *n* = 3 individual sensors. Groups were compared by a two-sided unpaired Student’s *t*-test. *P* = 0.0057. **d**, The r.m.s.d. of IAA in reference to two initial poses of IAA: in the vestibule and in the binding chamber. The rows of the matrix correspond to two PIN8–IAA simulation systems (with IAA starting in the vestibule versus the binding chamber). The columns correspond to IAA in individual monomers of each simulation repeat. Points are coloured according to the time progression of simulations, with a burgundy rhombus representing the starting ligand pose. Structures show the initial binding poses of IAA in both simulation systems, with IAA carbon atoms in orange, PIN8 scaffold domain residues in purple and elevator domain in red. The arrow indicates the dominant ligand movement observed during simulations. Figure created with PyMOL v5.0.4, Molecular Graphics System (Schrödinger, LLC). Source data

**Extended Data Fig. 5:**
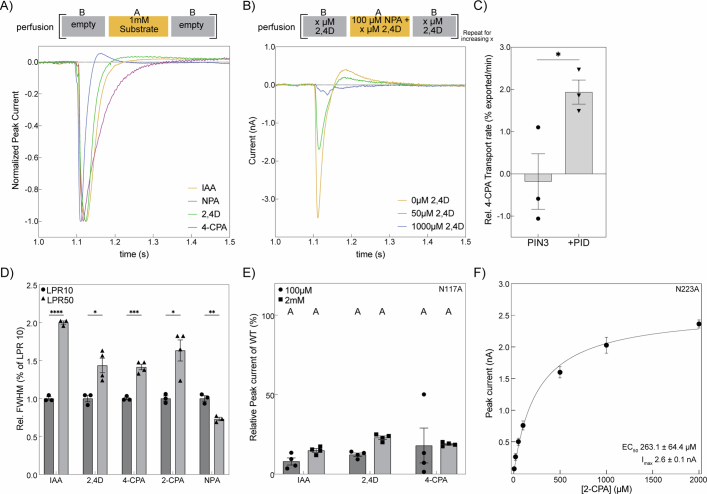
Transport of phenoxyacetic acids and identification of critical residues for Substrate interaction. **a**) Normalized representative current traces elicited by 1mM of the substrates. The perfusion protocol is shown on the top. **b**) Raw current traces of three representative solution exchange experiments used for *K*_*D*_ determination of 2,4-D. For clarity the graph only shows three of the concentrations used to obtain the *K*_*D*_. The perfusion protocol is shown on the top. **c**) Relative transport rates of [^3^H]-4-CPA export by PIN3 from oocytes in the presence (▼) or absence (•) of PID. Initial internal 4-CPA concentration was 1 μM. Data points are mean ± SE transport rates using oocytes collected from different females. Groups were compared by a two-sided unpaired Student’s t-test (PIN3 versus PIN3 + PID, * P = 0.0.4101. **d**) Full width half maximum (FWHM) from the Peak current response determined by SSM electrophysiology on the wild-type PIN8 proteoliposomes of LPR10 (•) and LPR50 (▲) derived from 2mM of indicated substrate. Data are mean ± s.e.m. of n = 3 individual sensors. Groups were compared by a two-sided unpaired Student’s t-test (IAA_LPR10_ versus IAA_LPR50_: **** P < 0.0001; 2,4-D_LPR10_ versus 2,4-D_LPR50_: * P = 0.0148; 4-CPA_LPR10_ versus 4-CPA_LPR50_: *** P = 0.0003; 2-CPA_LPR10_ versus 2-CPA_LPR50_: * P = 0.0127; NPA_LPR10_ versus NPA_LPR50_: ** P = 0.0032). **e**) Relative Peak current response compared to WT elicited by 100μM (•) or 2mM (■) of indicated substrates using SSM electrophysiology on PIN8 N117A proteoliposomes. Data are mean ± s.e.m. of n = 3 individual sensors. There are no significant differences between the substrates at both concentrations used (One way ANOVA p = 0.3). Letters indicate the same significance level. **f**) Peak current response as a function of [2-CPA] determined by SSM electrophysiology on PIN8 N223A proteoliposomes. A Michaelis–Menten model is fit to describe kinetics (${r}_{2-{CPA}}^{2}$ = 0.98; ${{EC}}_{50}^{2-{CPA}}$ 263 ± 64 μM; ${I}_{\max }^{2-{CPA}}$ 3 ± 0.1 nA; data points represent mean ± s.e.m.; n = 3 individual sensors). Source data

To explore chemical determinants of phenoxyacetic acid herbicides and to define crucial interactions within the binding chamber of PIN8, we measured binding affinity (*K*_D_) for a set of derivative compounds based on their competition with the inhibitor NPA. Specifically, we used the SSM assay to assess inhibition of the strong binding current produced by NPA (Extended Data Figs. 1 and 5b; ref. ^12^). To start, we analysed the known herbicides 2,4,5-trichlorophenoxyacetic acid (2,4,5-T), 2-methyl-4-chlorophenoxyacetic acid (MCPA) and 4-CPA in addition to 2,4-D, all of which share three critical features: the carboxylate group, the aryl ether group and a 4-Cl group (Fig. 2a,b, dark grey bars). Compared to IAA, we find that 4-CPA binds with significantly lower affinity (~8-fold, *P* < 0.05) (Fig. 2a and Extended Data Fig. 1a,i) confirming recent results for this compound^9^. The binding affinity of 2,4-D is similar to that of IAA, whereas MCPA binds with intermediate affinity (Figs. 1a and 2a and Extended Data Fig. 1a,d–f,i). Of all the substrates tested, 2,4,5-T binds with the highest affinity (Fig. 2a and Extended Data Fig. 1b). These results show that the number of substitutions in the phenyl ring, their position, chemical identity and influence on the π-orbital electrons impact substrate binding.

To characterize the impact of substitutions in the phenyl ring on substrate affinity, we investigated several compounds showing substitutions at different positions and with different chemistries. We find that phenoxyacetic acid, which lacks any substitutions at the phenyl ring, binds with significantly lower affinity (*K*_D_ ≈ 500 µM) (Fig. 2a,b and Extended Data Fig. 1j), indicating that chloride substitutions in the ring increase substrate affinity. Next, we determine that 3-(2,4-dichlorophenyl)-propionic acid binds with higher affinity than 2,4-D, suggesting that the aryl ether is not important (Fig. 2a,b and Extended Data Fig. 1c,d). 2-Chlorophenoxyacetic acid is bound with high affinity, comparable to that of IAA and 2,4-D (Fig. 2a,b and Extended Data Fig. 1a,d,e), suggesting that substitution at the C2 position on the phenyl ring is more important than at the C4 position (compare with 2-chlorophenoxyacetic acid versus 4-CPA). Specificity for chloride substitutions is evaluated by testing 2-methylphenoxyacetic acid (with a methyl group at C2), 4-methylphenoxyacetic acid (with a methyl group at C4) and 2,4-dimethylphenoxyacetic acid (with a methyl group at C2 and C4) (Fig. 2a,b and Extended Data Fig. 1g,h,k). Corresponding *K*_D_ values indicate that methyl groups produce lower affinity than chloride. Methyl groups in both C2 and C4 positions produce higher affinity than at C2 alone, whereas a methyl group at C4 alone produces even lower affinity than phenoxyacetic acid. This again shows that the C2 position is more important for substrate affinity than C4.

The ability of PIN8 to adopt different conformations during structure determination with 2,4-D suggested it would be possible to capture the key conformations representing a full transport cycle in a single cryo-EM sample. For this, we tried 4-CPA as a substrate which was recently found to be an important member of this group of herbicides with very low affinity associated with PIN-mediated export^12^. SSM measurements indicate that 4-CPA elicits NPA-sensitive current responses in PIN8 that have higher EC_50_ (800 ± 200 µM) and *K*_D_ (200 ± 100 µM), consistent with a lower affinity for 4-CPA relative to 2,4-D and IAA (Fig. 2c and Extended Data Fig. 1a,d,i). To establish that 4-CPA is also transported by PIN8, we compared the width of SSM-based current responses of IAA, 2,4-D and 4-CPA to that of the non-transported inhibitor NPA (Extended Data Fig. 5a,d)^21^. For IAA, 2,4-D and 4-CPA, the peak widths vary with lipid-to-protein ratio (LPR), consistent with transport of these compounds (Fig. 1b,c and Extended Data Figs. 2 and 5c). By contrast, peak width does not change with LPR for NPA, consistent with an inhibitor that produces a binding current but is not transported^21^.

**Fig. 3:**
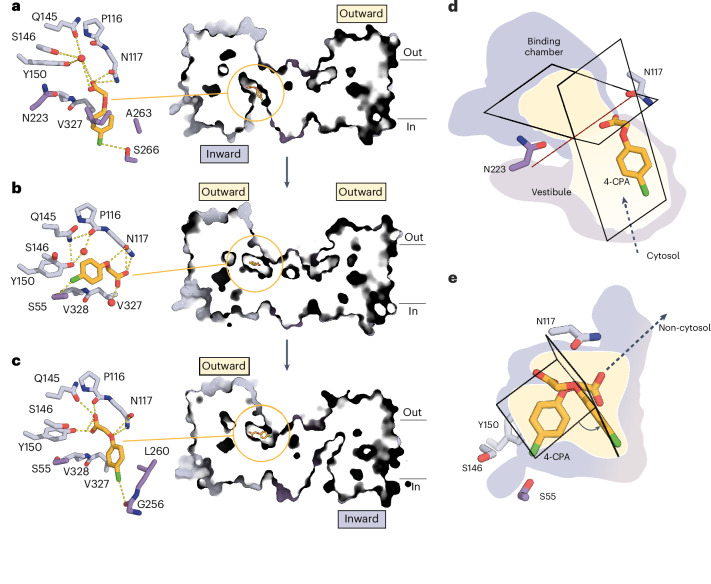
4-CPA structure and transport cycle. **a**, Close-up view (left) and cut-away view of surface presentation (right) of the asymmetric PIN8 structure bound 4-CPA in the prebinding state of the inward monomer. **b**, Close-up view (left) and cut-away view of surface presentation (right) of the symmetric PIN8 structure bound 4-CPA in the binding state of the outward monomer. **c**, Close-up view (left) and cut-away view of surface presentation (right) of the asymmetric PIN8 structure bound 4-CPA in the partly release state of the outward monomer. Residues belonging to the transporter domain highlighted in light violet and the scaffold domain in violet. Ligands are coloured orange. **d**, Overlay of 4-CPA prebinding position (PDB 9G0X and EMD-50951) in the inward monomer. The inward-facing pocket is divided into the binding chamber (light violet) and a vestibule (grey). The red broken line running from Asn117 to Asn223 defines the boundary between vestibule to binding chamber. **e**, Overlay of 4-CPA binding (PDB 9G0Z and EMD-50952) and partly released (PDB 9G10 and EMD-50953) position in the outward monomer. Both structures are superposed on the scaffold domain. The superposition shows that in either state, Asn117 retains its interaction with carboxyl and arylether groups. The planes define the position of the ring systems of NPA, IAA and 4-CPA within the vestibule and binding chamber in the inward monomer and outward facing monomer. Figure created with PyMOL v5.0.4, Molecular Graphics System (Schrödinger, LLC).

**Extended Data Fig. 6:**
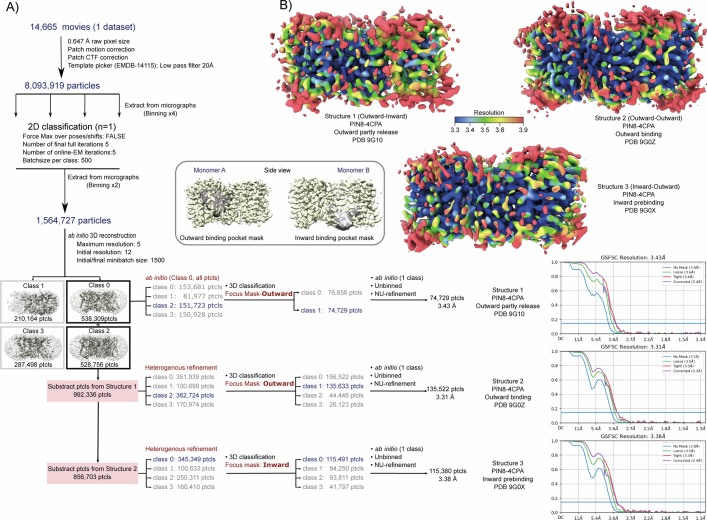
Image processing and reconstruction for 4-CPA bound PIN8. **a**) Workflow of image processing and 3D reconstruction in cryoSPARC, including a motion corrected micrograph from Titan Krios microscope using a K3 detector. **b**) Sharpened density map from the final non-linear refinement colored by local resolution. Corrected curve of the global Fourier shell correlation (FSC) indicates 3.43 Å (Structure 1), 3.31 Å (Structure 2), 3.38 Å (Structure 3) based on the 0.143 gold-standard criterion. The cryo-EM experiment with this sample was repeated 5 times with data collection 1 time. Figure created with Chimera v1.15 (ref. ^28^).

**Extended Data Fig. 7:**
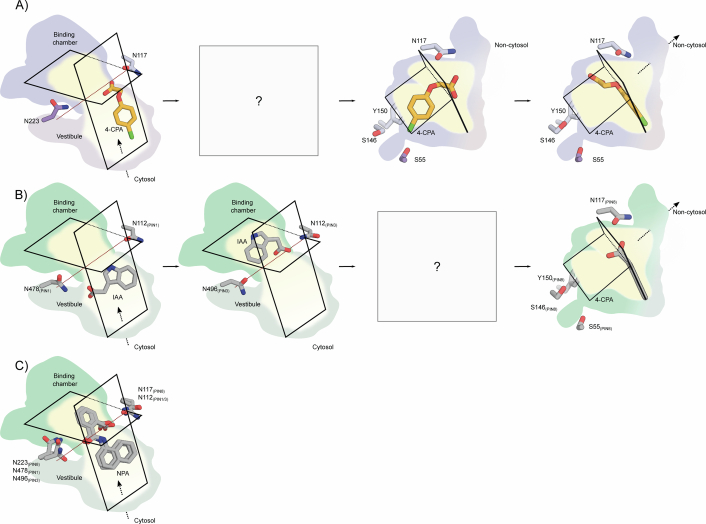
Substrate binding and interaction. Structures are superposed on the scaffold domain. The inward-facing pocket is divided into the binding chamber and a vestibule. The red broken line running from Asn117 to Asn223 defines the boundary between vestibule to binding chamber. (**a**) 4-CPA binding position and poses in the prebinding in the PIN8-inward conformation, binding and partly release in the PIN8-outward conformation. Square with question mark represents the missing state. (**b**) IAA binding position and poses in the prebinding in the PIN1-inward conformation, binding in the PIN3-inward conformation, and partly release in the PIN8-outward conformation. Square with question mark represents the missing state. (**c**) Overlay of NPA binding poses from PIN8, PIN1, PIN3. NPA binds in both the binding chamber and the vestibule and forms hydrogen bonds to both Asn117 and Asn223. The planes define the position of the ring systems of NPA, IAA and 4-CPA within the vestibule and binding chamber in the inward monomer and outward facing monomer. Figure created with PyMOL v5.0.4, Molecular Graphics System (Schrödinger, LLC).

Analysis of cryo-EM images of PIN8 with 4-CPA produced three independent structures that reveal five distinct conformations coexisting within a single sample (Extended Data Figs. 6 and 7a and Supplementary Table 1). These structures comprise both symmetric (OF/OF) and asymmetric (OF/IF) dimers with individual monomers adopting a range of different conformations representing both 4-CPA bound and empty states. By focusing on the ligand binding site for 3D classification, we are able to resolve five unique states: inward-facing empty (IF_empty_ at 3.4 Å), inward-facing with 4-CPA in a prebinding site in the vestibule (IF_4CPA-prebinding_ at 3.4 Å), outward-facing with 4-CPA in the binding pocket (OF_4CPA-bound_ at 3.3 Å), outward-facing with 4-CPA in a partly released state (OF_4CPA-partlyrelease_ at 3.3 Å) and outward-facing empty (OF_empty_ at 3.3 Å) (Fig. 3a–c, Extended Data Fig. 4b–d and Supplementary Video 1).

These structural data reflect the transitions of PIN8 and its substrate during the transport cycle (Fig. 3a–c and Extended Data Fig. 4b–d). In IF_4CPA-prebinding_, 4-CPA is located in the vestibule of PIN8 in a position similar to 2,4-D as described above: the carboxyl group is coordinated by Asn117, while a water molecule mediates contact to Gln145 and Tyr150 in the binding chamber. The 4-Cl atom is coordinated by Ser266 (Fig. 3a and Extended Data Fig. 4b). OF_4CPA-bound_ shows 4-CPA fully engaged with the binding chamber (Fig. 3b and Extended Data Fig. 4c). Here the carboxyl group of 4-CPA is coordinated by Asn117, and its charge is stabilized by the two dipoles of the crossover; the 4-Cl atom is now coordinated by Ser55. It is worth noting that the phenyl ring in OF_4CPA-bound_ is bound deeper in the binding chamber relative to IF_4CPA-prebinding_, suggesting that substrate recognition is initiated by binding of the carboxylate group followed by flipping and nestling of the phenyl ring into this chamber (Fig. 3d). OF_4CPA-partlyrelease_ shows that steps of substrate release are similar to those of binding, but in reverse. In the transition from OF_4CPA-bound_ to OF_4CPA-prerelease,_ the phenyl ring is solvated and flips out of the chamber, while the carboxyl maintains its interaction with Asn117 (Fig. 3c,e and Extended Data Fig. 4d). Together, these five structures allow us to follow the substrate as it transits through PIN8, highlighting various substrate poses and their transient and changing key interactions with the protein during transport.

To further explore substrate recognition between the vestibule and the binding chamber, we performed molecular dynamics simulations of PIN8 IF/IF. Modelling chlorinated compounds in molecular dynamics simulations is challenging due to the strong electronic effect associated with the halogen bonds^22^. For this reason, we used the native substrate IAA in two starting poses to examine dynamics and interactions by molecular dynamics: either IAA in the vestibule or IAA in the binding chamber. At the start of each of three independent simulations, IAA was placed symmetrically in both monomers and run for a duration of 550 ns, adding up to a total of 1,650 ns for each configuration.

Although no large-scale conformational changes were observed in the protein, there were notable changes in atomic interactions and ligand pose of IAA. Specifically, the IAA substrate initially placed in the vestibule flips into the binding chamber of IF in two thirds of the examined simulation runs (Fig. 2d). Conversely, IAA that is initially placed within the binding chamber remains bound and shows less movement, presumably due to a strong network of stabilizing interactions involving the IAA carboxyl group and polar oxygens on Asn117 and Tyr150. The rapid ‘flip’ of IAA from vestibule to the binding chamber, but not in reverse, supports that this movement is directed by the architecture of the binding site and associated with the transport process. Considering the molecular similarity between IAA and its analogues, the same flip movement might also be important for herbicides that are transported by PIN8 via the binding chamber.

**Fig. 4:**
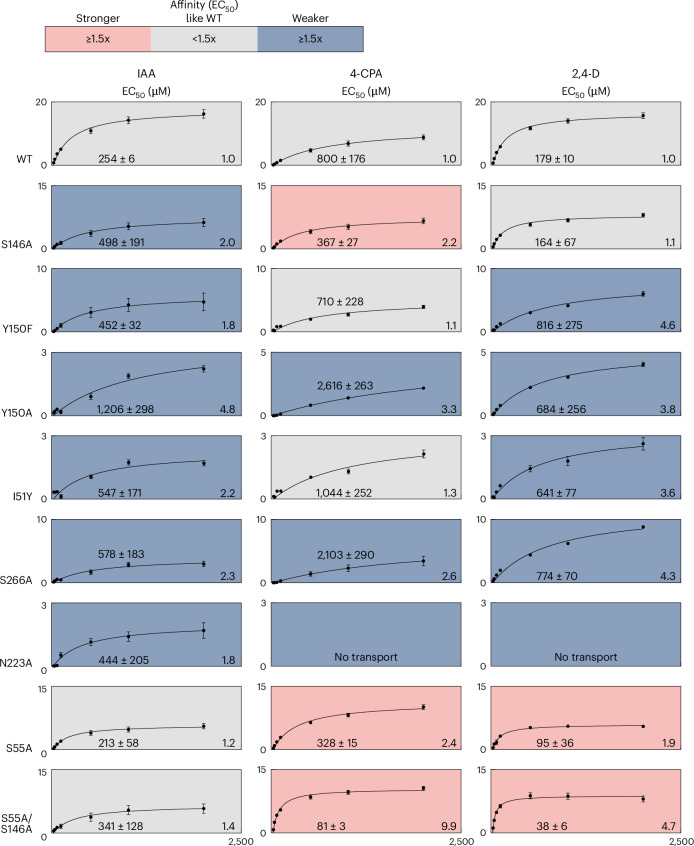
Identification of key residues required for substrate binding. EC_50_ of peak current response as a function of the substrates indicated derived from the full measurements. A Michaelis–Menten model is fit to describe kinetics (Supplementary Table 4). Red colour indicates ≥1.5 times higher affinity than wild type (WT); blue colour indicates ≥1.5 times lower affinity than WT; grey colour indicates <1.5 times change from WT affinity. No transport means that a Michaelis–Menten model could not be fit. The *x* axis unit is µM, *y* axis unit is (nA) and the fold-change differences to the respective EC_50_ of wildtype PIN8 is indicated in the bottom right corner of each graph. Data points represent mean ± s.e.m. For individual *n* values, see Supplementary Table 4. Source data

To identify molecular determinants of PIN8 substrate recognition based on the structural and molecular dynamics data, we examined the IF_4CPA-prebinding_ structure to identify key residues and used SSM electrophysiology to compare binding of 4-CPA, IAA and 2,4-D to single-site mutants (Fig. 4 and Extended Data Fig. 5e,f). It is well established that Asn117 is critical for coordination of the carboxylate during IAA transport^12^. Accordingly, the N117A mutation abolishes the current response for both 2,4-D and 4-CPA (Extended Data Fig. 5e). Ser146 and Tyr150 interact with the carboxylate via a water molecule (Figs. 1d and 3a; ref. ^12^). Consistent with this indirect coordination, S146A and Y150F mutations reduced substrate affinity to IAA slightly (~2-fold), while Y150A reduced substrate affinity further (3.3- to 4.8-fold) for all three compounds. Introduction of a bulky side chain with the I51Y mutation reduces substrate affinity, likely by hindering substrate entry into the prebinding state. Ser266 directly coordinates the 4-Cl residue (Figs. 1d and 3a), and the S266A mutation reduces affinity for all substrates.

The structures suggest that Asn223 is a critical residue that delineates the vestibule from the binding pocket in the inward conformation. In contrast to previous work on PIN1^13^, we find that the N223A mutant reduces affinity for IAA only slightly but completely abolishes binding of 2,4-D and 4-CPA. This result suggests that once the substrate has flipped from the prebinding position into the binding pocket, Asn223 is required to keep the substrate in place (Extended Data Fig. 5f). In the outward open substrate-bound state Ser55 is near the 4-Cl group (Fig. 3b,e). We reasoned that mutating Ser55 to an alanine would accommodate a better fit of 2,4-D and 4-CPA as well as support the more hydrophobic nature of the halogenated phenyl ring. Indeed, the S55A mutant shows higher affinity (1.9- to 2.4-fold) for the herbicides, whereas the affinity for IAA remains unchanged. To further support the notion that increased hydrophobicity and size of the binding pocket size can selectively improve binding of the chlorinated auxin herbicides, we tested the double mutant S55A/S146A (Fig. 4), which increases affinity towards chlorinated auxin herbicides even further (4.7- to 9.9-fold), again with no effect on affinity of the natural auxin IAA. Taken together, the results suggest that by combining chemical variability in substrates and protein mutagenesis, it will be possible to design novel auxins and engineer resistance to these compounds by a structure-guided approach.

## Discussion

We have shown that synthetic auxins are transported by PIN proteins both in planta and in vitro and have explored the biochemical, structural and chemical parameters that govern transport. Specifically, we have used cryo-EM to describe changes in substrate binding as the protein undergoes conformational changes associated with transport. We have used structure-guided mutagenesis to evaluate principal components of the binding pocket that recognize and interact with substrates and have used chemical biology to elucidate substrate determinants of binding affinity. We have also assessed the dynamics and directionality of substrate binding using molecular dynamics simulations. Our work highlights key chemical and structural elements of both herbicides and auxin transporters that can be adjusted to augment or prevent herbicide distribution in plants.

Structural analysis of transport under turnover conditions has been demonstrated for primary active transporters^23,24^. This has been facilitated by their relatively slow turnover rate and by the ability to use ATP to initiate the transport cycle before flash freezing of cryo-EM samples. For uniporters, steady state conditions can be achieved by adding subsaturating concentrations of substrate, thus allowing a population of proteins to adopt all conformational states associated with the transport cycle in proportions that reflect the kinetics of this cycle. In the case of 4-CPA and PIN8, the structures provide a nearly comprehensive mapping of molecular interactions between a PIN transporter and an auxin herbicide as it transits across the membrane (Extended Data Fig. 7). The structural data and the molecular dynamics simulations support that transport is a multi-step process with auxin first recognized in the vestibule before nestling into the binding pocket. Occupation of this pocket induces the change to the outward state, where the binding steps are reversed. An occluded state with substrate trapped in the binding pocket has not been observed, suggesting that it is a short-lived, unstable state, at least in the presence of 4-CPA.

Binding of phenoxyacetic acid herbicides to PINs is very similar to the natural substrate, IAA (Extended Data Fig. 7). The substrates enter the vestibule, and the carboxylate is coordinated directly by Asn117 and indirectly by Tyr150, Ser146 and Gln145 via a water molecule. These findings are consistent with previous work in which we determined Asn117 to be critical for the coordination of IAA’s carboxylate and for transport activity in PIN8^12^. In the current work, 2,4-D is seen in a prebinding position with its carboxyl group interacting with Asn117 and with crossover dipoles from the nearby half-helices neutralizing its negative charge; like IAA, 2,4-D is transported with an EC_50_ of ~200 μM, and the N117A mutation abolishes function (Figs. 1a and 4 and Extended Data Fig. 5e). Although this general location of 2,4-D is similar to the IAA position in the previously published structure of PIN1, the pose is different and closer to that of IAA in the previously published structure of PIN3^12,13,15^. Based on SSM analysis, the 4-Cl group and its interaction with Asn223 is critical. We show that Ser55 and Tyr150 play the critical role in the binding pocket. Removing the polar group of Ser55 accommodates a better fit of the halogenated substrate, while the non-halogenated ring of IAA is accommodated with no changes in affinity. Addition of Cl groups to the benzyl ring reduces the electron density of the π-electrons over the benzyl ring^25^ until a π-configuration similar to the indole ring is achieved. It is worth noting that this configuration is not stabilized by methyl groups in the ring (Fig. 2a).

**Extended Data Fig. 8:**
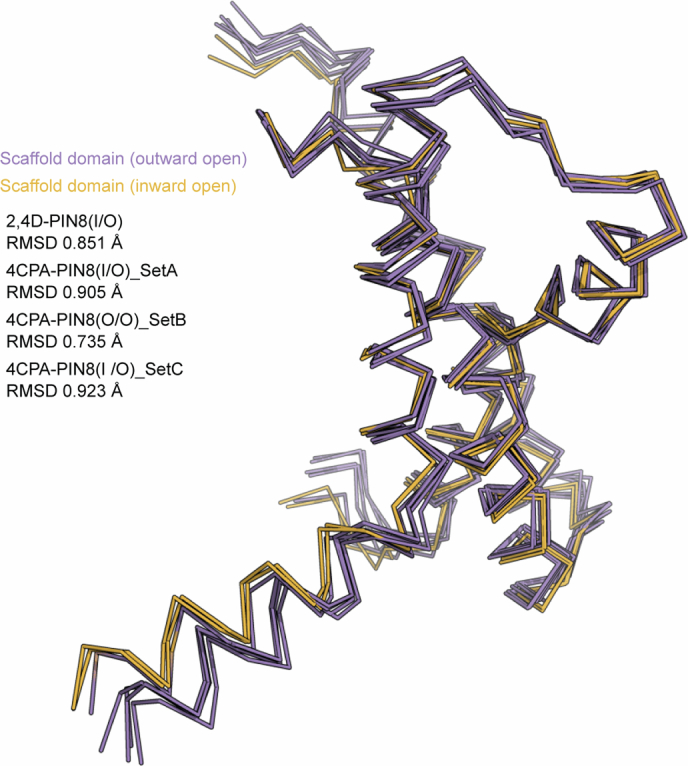
Scaffold domain during the transport. Superposition of scaffold domain in different transport states show that the scaffold domain does not change during transport. Figure created with PyMOL v5.0.4, Molecular Graphics System (Schrödinger, LLC).

Like many other transporters using the elevator mechanism, PINs assemble into dimers, and this assembly has been hypothesized to provide potential for cooperative action during transport. Here we show that, at least for PIN-FORMED auxin transporters, the conformation of protomers within the dimeric assembly are uncorrelated. This observation is inconsistent with cooperativity; rather, it suggests that each protomer operates independently. Regarding the elevator mechanism, superposition of transport and scaffold domains of all the structures show that PIN transport involves rigid body movements, without local changes in these individual domains (Figs. 1d and 3a–c and Extended Data Figs. 7 and 8); transition from inward to outward states involves a translation of the transport domain in a direction normal to the membrane plane while the scaffold domain remains fixed within this plane and anchored to its dimeric partner molecule.

In conclusion, we have shown that phenoxyacetic acid auxin herbicides are transported by PINs. We have used cryo-EM to study PIN8 and have captured five key states during transport of the herbicide 4-CPA. We have elaborated on the mechanism of substrate transfer from the vestibule to the binding chamber through molecular dynamics simulations and have used chemical biology to explore the determinants of substrate specificity. This structural analysis elucidates the steps of substrate binding and release, provides a model to explain how chemical diversity of synthetic auxins can be accommodated and indicates that dimeric transporters with a crossover fold can operate asymmetrically with an apparent lack of cooperativity. Future research will show whether other auxin herbicides are also PIN substrates and whether the determinants of substrate affinity can be generalized to include other halogenated auxin herbicides such as dicamba or picloram^8^. In this way, our elucidation of the determinants of substrate affinity may be used to identify novel auxin herbicides with higher systemic mobility for use in agriculture.

## Methods

### Solid supported membrane-based electrophysiology assays

SSM, using a SURFE2R N1 from Nanion Technologies, was conducted as described^12^. In brief, soy polar lipid mix (38% phosphatidyl choline, 30% phosphatidyl ethanolamine, 18% phosphatidyl inositol, 7% phosphatidic acid and 7% other soy lipids) and 1-palmitoyl-2-oleoyl-*sn*-glycero-3-phosphocholin were purchased from Avanti. Liposomes were prepared in Ringer solution without Ca^2+^ (115 mM NaCl, 2.5 mM KCl, 1 mM NaHCO_3_, 10 mM HEPES pH7.4, 1 mM MgCl_2_) and homogenized using a Lipsofast (Avestin) with a 400 nM pore size. Triton X-100 was added to the liposomes to a final concentration of 1% (*v*/*v*). Protein was added to liposomes to a calculated lipid-to-protein ratio (LPR) of 10 unless stated otherwise. The detergent was removed using 400 mg ml^−1^ Bio Beads (BioRad) overnight at 4 °C in a rotary shaker. Proteoliposomes were frozen in liquid nitrogen and kept at −80 °C until use. Proteoliposomes were diluted 1:5 in Ringer solution without Ca^2+^, sonicated five times and then applied to the sensors by centrifugation (30 min, 3,000 *g*, 4 °C). Non-activating buffer was Ringer solution without Ca^2+^ as described unless specified otherwise, and activating buffer contained the substrate of interest. In most instances we used a single-solution exchange experiment. In this case proteoliposomes, immobilized on the supported membrane, are kept in non-activating buffer as specified. At the beginning of the experiment the non-activating buffer is replaced with fresh identical non-activating buffer, and after 1 s, activating buffer (same buffer containing substrate) is added. After another second, the buffer is replaced with non-activating buffer again. Current response is recorded throughout the entire 3 s. For competition or inhibition, the respective compound was present in non-activating and activating solutions. Each experiment was performed on at least two individual sensors. On each sensor each measurement consists of three technical replicates where the mean is calculated. *K*_D_ values were determined as described previously^12^. Briefly, the binding current induced by 100 µM NPA was recorded in the presence of different concentrations of the substrate under investigation ranging between 0 µM and 1,000 µM present in the activating solution and the non-activating solution (Extended Data Fig. 5b). Representative traces are shown for 0, 50 and 1,000 µM 2,4-D (Extended Data Fig. 5b). The maximum peak current was then plotted as a function of the concentration of the substrate under investigation and plotted against the degree of inhibition and fitted with a one-site binding model using GraphPad Prism V10.4.1 The half maximal concentration corresponds to the *K*_D_ of the substrate under investigation.

### Oocyte efflux assays

Oocyte efflux experiments were carried out as described^12,20,26^. Briefly, oocytes were injected with 150 ng transporter complementary RNA without or with 75 ng kinase cRNA. [^3^H]-IAA (25 Ci mmol^−1^) and [^3^H]-4-CPA (37.7 Ci mmol^−1^) was purchased from RC Tritec. [^3^H]-2,4-D (25 Ci mmol^−1^) was purchased from ARC. Oocytes were injected with substrate to reach an internal concentration of 1 µM, corresponding to 100%. Residual radioactivity was determined for each individual oocyte by liquid scintillation counting after the time points indicated and are expressed relative to the initial 100%. Each time point represents the mean and s.e. of ten oocytes. To calculate the relative transport rate in percentage per minute, linear regression was performed. Each data point in Fig. 1b,c and Extended Data Fig. 2b,e represents the transport rate of one biological replicate using oocytes collected from different *Xenopus* females.

### Stem assays

*Arabidopsis* stem transport assays were carried out as described^20^. Briefly, 2 cm stem sections were cut above the rosette of 5-week-old plants and placed in inverted orientation into auxin transport buffer containing 500 pM IAA, 1% (wt/vol) sucrose, 5 mM 2-(*N*-morpholino)ethanesulfonic acid, pH 5.5 with or without 100 μM NPA. After 2 h the stems were transferred into auxin transport buffer containing 417 nM [^3^H]-IAA or [^3^H]-2,4-D as a substrate in the absence and presence of NPA. After 2 h, 5 mm segments were dissected, and the substrate was quantified using a liquid scintillation counter.

### Cryo-EM sample preparation

The protocol for expression and purification of AtPIN8 protein (Uniprot: Q9LFP6) in lauryl maltose neopentyl glycol was described previously^12^. All point mutants were generated using Q5 Site-Directed mutagenesis kit (New England Biolabs). The C-flat Holey Carbon grids (CF-1.2/1.3, Cu-300 mesh) were glow-discharged for 45 s at 15 mA in a GloQube Plus (Quorum). The 2,4-D sample was prepared by mixing freshly purified PIN8 at 9.4 mg ml^−1^ in G-Buffer (50 mM Tris pH 7.5, 0.15 M NaCl, 0.0006% lauryl maltose neopentyl glycol) with 2,4-D (dissolved in DMSO) to reach a ~15 mM final concentration. After 1 h of incubation on ice, the sample was centrifuged at 16,000 *g* for 15 min before blotting to remove precipitation. A drop of 4 µl of each sample was applied to the glow-discharged grids, blotted with a Vitrobot Mark IV (ThermoFisher Scientific) using the following settings (temperature 4 °C, 100% humidity, blot time of 4 s, blot force −1) and vitrified in liquid ethane. For 4-CPA, after mixing protein (8 mg ml^−1^) with 25 mM 4-CPA (dissolved in DMSO) on ice, sample was rapidly applied on the grid without centrifugation. We did not accurately estimate the incubation time during blotting, but the incubation window for the collected grid is between 10 and 20 min.

### Image collection and data processing

A Titan Krios G3i microscope (ThermoFisher Scientific) operating at 300 kV and equipped with a BioQuantum Imaging Filter (energy slit width of 20 eV) with a K3 detector (Gatan) was used to create the movies. The datasets were acquired using automated acquisition EPU Software at nominal 130,000 magnification corresponding to a physical pixel size 0.647 Å. For all datasets, the movies were saved in super-resolution pixel size and binned two times in EPU Software back to the nominal pixel size.

Micrographs were imported into cryoSPARC and processed for patch motion correction, patch contrast transfer function estimation and particle picking with blob picker v4.5.1 (ref. ^27^). After several rounds of particle cleaning, an initial preliminary volume map was used to create templates for template picking.

From a full dataset of 2,4-D PIN8 with 8,473 movies, template picking provided a total of 3,173,516 particles. The full stack of particles was divided into four smaller particles stack to reduce computational time. After 2 rounds of 2D classifications, manually selected 2D classes from each stack were combined, yielding a total of 875,481 particles, re-extracted in a box size of 416 pixels (Fourier-crop to box size 208) and divided into two particles stack.

The initial particle stack was processed independently three times and recombined after 2 rounds of 2D classifications, providing a total of 662,418 particles, serving as input for ab initio model reconstruction. Of four ab initio models, the best representative ab initio model was used as a ‘good’ template volume. A ‘bad’ template volume was generated by low-pass filtering the good template volume to 40 Å. The corresponding particles stack from good ab initio classes (209,920 particles) were sorted with rounds of heterogeneous refinement using 3 good and 3 bad template volumes and processed independently 4 times. The good particle stack from each individual heterogenous refinement were recombined, resulting in a total of 281,146 particles. Following an ab initio model reconstruction, particles from the good class were re-extracted and were used for non-uniform refinement with C1 symmetry imposed, which resulted in a global 3.5 Å resolution map.

The entire 4-CPA PIN8 dataset comprised 14,655 movies, and template-picking yielded a total of 8,093,919 particles. The full stack of particles was divided into four smaller subsets. After 2D classification, selected 2D class particles were combined, resulting in a total of 1,564,727 particles. After two rounds of ab initio model reconstruction, two classes with clear transmembrane helices were selected for further processing. Preliminary model fitting into reconstructed ab initio volume suggested the presence of an asymmetric (class 0) and symmetric (class 2) configuration of each monomer in the PIN dimer.

Particles belonging to the ab initio class in which each monomer adopted an outward–inward configuration were further processed by a second ab initio reconstruction and focus-3D classification with a spherical mask (15 Å, dilation radius 2 Å, soft padding width 5) centring around the outward binding pocket in the outward monomer. Particles from each class were further used for non-uniform refinement with C1 symmetry imposed, and only one class resulted in a global 3.4 Å resolution map with well-defined ligands density in the outward binding pocket (map 1).

To avoid the recycling of particles belonging to the first map, particles belonging to map 1 were subtracted from the initial particles stack from the main ab initio class. The remaining stack (992,336 particles) was subjected to heterogenous refinement job using volume from the previous main ab initio class as template. Similarly, particles belonging to the class in which each monomer adopted an outward–outward configuration were processed using a focus-3D classification with a spherical mask centring around the outward binding pocket in one outward monomer. Particles from each class were further used for non-uniform refinement with C1 symmetry imposed, and only one class resulted in a global 3.3 Å resolution map with well-defined ligands density in the outward binding pocket (map 2). The same strategy was used with a focus mask centring around the inward binding pocket. The isolated particles stack is further refined to 3.4 Å resolution map (C1 symmetry) with well-defined ligand density in the inward binding pocket (map 3).

### Model building and refinement

The published apo-PIN8 structure (Protein Data Bank (PDB) accession code 7QP9) served as initial model for docking into the map for 2,4-D and 4-CPA using Chimera^28^. Molecular dynamics-based geometry fitting using molecular dynamics flexible fitting^29^ was carried out by Namdinator^30^. After this, models were manually adjusted by iterative model building in Coot 0.9.6 combined with real space refinement using Phenix 1.19.2, initially with an Amber force-field molecular dynamic refinement^31^. The coordination of lipids and the ligand 2,4-D, 4-CPA and DMSO was obtained from GRADE v1.6.0 (https://grade.globalphasing.org). Structure geometry was validated by monitoring with MolProbity 4.5.2, CaBLAM and Ramachandran-Z analysis (Rama-Z)^32–34^. Statistics of the model refinement can be found in Supplementary Table 1.

### Molecular dynamics system preparation

Parameters for the IAA substrate were prepared using CGenFF 2021.1 (refs. ^35,36^). The inwards-open conformation of PIN8 (PDB accession code 7QPC) was processed in Maestro 2021.4 (ref. ^37^). The complete N-terminus was modelled as charged (NH3^+^), while the truncated C-terminus was capped with an amide group. We omitted rebuilding the 40-residue-long missing loop (spanning residues 165–205) as it was presumed to be disordered; instead, the backbone edges at residues 164 and 206 were modelled as uncharged COOH and NH_2_, respectively. All histidines in both monomers were modelled as neutral: His9, His30, His237, His253 and His339 with a proton bound to the Nδ atom, and His94 with a proton on the Nε atom of the imidazole ring. A water molecule in the support site of each monomer (coordinated by Asp75, Lys79, Gln78, Gln145 and Gln320) was also included in the model. The PIN8 dimer was aligned with the *xy* plane using the Orientations of Proteins in Membranes database with Positioning of Proteins in Membranes 3.0 web server^38^ and with both N- and C-termini pointing in the positive direction of the Cartesian coordinate system.

IAA pose when bound to PIN8 was informed by the experimentally resolved PIN structures that include either the substrate (IAA) or an analogue inhibitor (NPA). IAA placement within the binding chamber was informed by the PIN3–IAA complex (PDB accession code 7XXB); IAA in the vestibule was aligned with 4-CPA in IF_4CPA-prebinding_ by pairwise alignment of C18_IAC_ and C7_4CPA_, N_IAC_ and C2_4CPA_, C_IAC_ and C1_4CPA_, and C7_IAC_ and C5_4CPA_.

The resulting PIN8–IAA dimer complex was placed in a 125 Å × 125 Å membrane consisting of 1-palmitoyl-2-oleoyl-*sn*-glycero-3-phosphocholin lipids using CHARMM-GUI version 3.7 (refs. ^39,40^). System solvation and neutralization using 0.15 NaCl was performed using Gromacs tools.

### Molecular dynamics simulation protocol

The modelled system was simulated using the GROMACS 2024.3 simulation software^41^. The molecular dynamics simulations were performed using the CHARMM36m force field (July 2022 release)^42,43^. The initial minimization was performed using a steepest-descent algorithm in 5,000 steps until the forces on each atom dropped below 1,000 kJ mol^−1^ nm^−1^.

The following six runs of short equilibrations were adapted from the CHARMM-GUI membrane equilibration protocol with a v-rescale thermostat^44^ on three temperature groups (applied to all equilibration steps) and semi-isotropic pressure maintained with a c-rescale barostat^45^ (applied from the third equilibration step onwards). The backbone position restraints were fixed at 1,000 kJ mol^−1^ nm^−2^, while the ligand, lipid phosphorus atom and lipid dihedral angle position restraints were progressively turned off. Protein side-chain position restraints were sequentially lowered to 100 kJ mol^−1^ nm^−2^.

To allow the ligand a longer time to find a favourable binding pose, we supplemented a 50-ns-long equilibration run in an NPT regime with unrestrained ligand and membrane lipids, protein backbone position restraints at 1,000 kJ mol^−1^ nm^−2^ and side-chain restraints at 100 kJ mol^−1^ nm^−2^.

The production simulations were run for 500 ns with a 2 fs time step and with an applied LINCS algorithm that constrained the covalent bonds with hydrogens^46^. V-rescale thermostat was applied to three temperature groups with a time constant of 1 ps and the temperature maintained at 310 K. C-rescale barostat maintained the semi-isotropic pressure at 1 bar with a time constant of 5 ps and compressibility of 4.5 × 10^−5^ bar^−1^ in both dimensions. The short-range electrostatic interactions were considered up to 1.2 nm cut-off, while the long-range electrostatic interactions were calculated using the particle mesh Ewald algorithm^47,48^. The cut-off for van der Waals interactions was set to 1.2 nm with a force-switch modifier applied from 1.0 nm. Both simulation systems (with IAA in the binding chamber and with IAA in the vestibule) were run in triplicates. As each simulation system consisted of a dimer, where each monomer and its ligand were considered to behave in an independent manner, the sampling of ligand–protein interactions was effectively doubled to 3 µs.

### Molecular dynamics simulation analysis

The molecular dynamics analyses were performed on the joint final equilibration run and the production run (550 ns for each repeat), as IAA was unrestrained in the final equilibration run and could therefore move away from its starting reference position. The root-mean-square deviation (r.m.s.d.) of IAA was calculated for ligand heavy atoms and with regards to two reference positions: IAA in the binding chamber and IAA in the vestibule. The calculations were performed using Gromacs 2024.3 analysis tools. Analysis of IAA was performed separately for each monomer.

### Statistical analyses

Statistical analyses were performed using GraphPad Prism 10.2. Parameters of the individual tests and results are shown in Supplementary Tables 1–3.

### Reporting summary

Further information on research design is available in the Nature Portfolio Reporting Summary linked to this article.

## Supplementary information

Supplementary Information Supplementary Tables 1–4.

Reporting Summary

Supplementary Video 1 Morph of captured and modeled structures of PIN8 with 4-CPA.

## Source data

Source Data Figs. 1, 2 and 4 and Extended Data Figs. 1, 2 and 5 Source data.

## Data Availability

Materials are available upon request. Atomic models have been deposited in the Protein Data Bank, and cryo-EM maps have been deposited in the Electron Microscopy Data Bank. 2,4-D inward prebinding (vestibule): PDB 9G0W and EMD-50950. 4-CPA inward prebinding (vestibule) (*I*_4CPA prebinding_/*O*_empty_): PDB 9G0X and EMD-50951. 4-CPA outward binding (*O*_4CPA binding_/*O*_empty_): PDB 9G0Z and EMD-50952. 4-CPA outward partly released (*O*_4CPA, partlyreleased_/*I*_empty_): PDB 9G10 and EMD-50953. Source data are provided with this paper.
