# Supplementary material for: Transport of phenoxyacetic acid herbicides by PIN-FORMED auxin transporters
Source: Nat Plants. 2025 Apr 22;11(5):1049–59. doi: 10.1038/s41477-025-01984-0 (PMC12095047; doi:10.1038/s41477-025-01984-0)
Supplement: Supplementary file 1 — Supplementary Tables 1–4. [file 41477_2025_1984_MOESM1_ESM.pdf]

nature plants

Supplementary information https://doi.org/10.1038/s41477-025-01984-0

Transport of phenoxyacetic acid herbicides by PIN-FORMED auxin transporters

In the format provided by the authors and unedited

Supplementary Table 1: Statistics for cryo-EM data collection, model refinement and validation

Cryo-EM data collection, refinement and validation statistics

	Dataset 1	Dataset 2		
	**Inward-Outward**<br>2,4-D PIN8<br>detergent (LMNG)<br>Prebinding state	**Ouward-Inward**<br>4-CPA PIN8<br>detergent (LMNG)<br>Partly release state	**Ouward-Outward**<br>4-CPA PIN8<br>detergent (LMNG)<br>Binding state	**Inward-Outward**<br>4-CPA PIN8<br>detergent (LMNG)<br>Prebinding state
**Data Collection and processing**				
Magnification	130,000	130,000	130,000	130,000
Voltage (kV)	300	300	300	300
Electron exposure ($e^{-}/Å^2$)	59.0	58.4	58.4	58.4
Defocus range (μm)	0.5-2.5	0.5-2.5	0.5-2.5	0.5-2.5
Pixel size (Å)	0.647	0.647	0.647	0.647
Symmetry imposed	C1	C1	C1	C1
Collected micrographs (no.)	8,473	14,665	14,665	14,665
Initial particle images (no.)	3,173,516	8,093,919	8,093,919	8,093,919
Final particle images (no.)	98,941	74,729	135,522	115,380
Map resolution (Å)*	3.54	3.43	3.31	3.38
**Refinement**				
Initial model used (PDB code)	7QP9	7QP9	7QP9	7QP9
Map sharpening B factor ($Å^2$)	-108.5	-103.2	-93.3	-94.4
Model composition				
non-hydrogen atoms	5,064	5,141	5,137	5124
Protein residues	640	644	644	642
Ligands	DLP: 1, CFA:1, DMS: 1	DLP: 2, A1IHP:1, DMS: 1	DLP: 2, A1IHP:1	DLP: 2, A1IHP:1
Waters	4	4	5	3
R.m.s. deviations				
Bond lenghts (Å)	0.002	0.003	0.003	0.003
Bond angles (deg)	0.565	0.573	0.564	0.649
Validation				
MolProbity score	1.60	1.68	1.70	1.39
Clashscore	7.46	7.51	7.15	5.65
Poor rotamers (%)	0.00	0.00	0.00	0.00
Rama-Z score (Whole/Helix/Loop)	1.18 / 1.32 / -1.26	1.63 / 1.69 / -1.41	1.48 / 1.68 / -1.80	-2.37 / -1.39 / -1.46
CaBLAM score (Outliers/Disfavored/Cα)	1.30 / 3.73 / 0.49	0.65 / 3.71 / 0.00	0.65 / 4.35 / 0.16	0.97 / 3.07 / 0.32
Ramachandran Plot				
Favored (%)	96.82	96.04	95.57	97.62
Allowed (%)	3.03	3.80	4.43	2.38
Disallowed (%)	0.16	0.16	0.00	0.00
Deposited model (PDB id)	9G0W	9G10	9G0Z	9G0X
Deposited map (EMDB id)	EMD-50950	EMD-50953	EMD-50952	EMD-50951

* Gold standard FSC with threshold of 0.143
DLP: 1,2-Dilinoleoyl-SN-Glycero-3-Phosphocholine
CFA: 2,4-Dichlorophenoxyacetic acid
A1IHP: 4-Chlorophenoxyacetic acid
DMS: Dimethyl sulfoxide

Supplementary Table 2: Results of the statistical analyses performed in Extended Data Fig. 1H and I. Groups were compared by a two-sided unpaired Student's t-test.

**Substrate**	**NPA**	**Segment**	**P value**
IAA	-NPA	WT^{Seg1} vs $pin1^{Seg1}$	0.0005
		WT^{Seg2} vs $pin1^{Seg2}$	0.001
		WT^{Seg3} vs $pin1^{Seg3}$	0.036
	+NPA	WT^{Seg1} vs $pin1^{Seg1}$	0.7145
		WT^{Seg2} vs $pin1^{Seg2}$	0.8337
		WT^{Seg3} vs $pin1^{Seg3}$	0.0909
2,4-D	-NPA	WT^{Seg1} vs $pin1^{Seg1}$	0.0042
		WT^{Seg2} vs $pin1^{Seg2}$	0.0781
		WT^{Seg3} vs $pin1^{Seg3}$	> 0.9999
	+NPA	WT^{Seg1} vs $pin1^{Seg1}$	0.1638
		WT^{Seg2} vs $pin1^{Seg2}$	0.4574
		WT^{Seg3} vs $pin1^{Seg3}$	0.4979

Supplementary Table 3: Results of the statistical analysis shown in Fig. 2A. One-way ANOVA followed by a Turkey´s multiple comparisons test ($p < 0.05$).

**Tukey's multiple comparisons test**	**P value**
IAA vs. 2,4-D	>0.9999
IAA vs. 4-CPA	<0.0001
IAA vs. MCPA	0.3977
IAA vs. 2-CPA	0.8412
IAA vs. PA	<0.0001
IAA vs. 2,4-DPP	>0.9999
IAA vs. 2,4,5-T	0.9995
IAA vs. 2-MPA	0.0013
IAA vs. 4-MPA	<0.0001
IAA vs. 2,4-DMPA	0.0826
2,4-D vs. 4-CPA	0.0002
2,4-D vs. MCPA	0.6967
2,4-D vs. 2-CPA	0.9808
2,4-D vs. PA	<0.0001
2,4-D vs. 2,4-DPP	0.9962
2,4-D vs. 2,4,5-T	0.9711
2,4-D vs. 2-MPA	0.0041
2,4-D vs. 4-MPA	<0.0001
2,4-D vs. 2,4-DMPA	0.2088
4-CPA vs. MCPA	0.0156
4-CPA vs. 2-CPA	0.0027
4-CPA vs. PA	0.0056
4-CPA vs. 2,4-DPP	<0.0001
4-CPA vs. 2,4,5-T	<0.0001
4-CPA vs. 2-MPA	0.9512
4-CPA vs. 4-MPA	<0.0001
4-CPA vs. 2,4-DMPA	0.1058
MCPA vs. 2-CPA	0.9993
MCPA vs. PA	<0.0001
MCPA vs. 2,4-DPP	0.2002
MCPA vs. 2,4,5-T	0.1158
MCPA vs. 2-MPA	0.2304
MCPA vs. 4-MPA	<0.0001
MCPA vs. 2,4-DMPA	0.9969
2-CPA vs. PA	<0.0001
2-CPA vs. 2,4-DPP	0.5929
2-CPA vs. 2,4,5-T	0.4132
2-CPA vs. 2-MPA	0.0538
2-CPA vs. 4-MPA	<0.0001
2-CPA vs. 2,4-DMPA	0.8334
PA vs. 2,4-DPP	<0.0001
PA vs. 2,4,5-T	<0.0001
PA vs. 2-MPA	0.0003
PA vs. 4-MPA	0.1258
PA vs. 2,4-DMPA	<0.0001
2,4-DPP vs. 2,4,5-T	>0.9999
2,4-DPP vs. 2-MPA	0.0005
2,4-DPP vs. 4-MPA	<0.0001
2,4-DPP vs. 2,4-DMPA	0.0333

2,4,5-T vs. 2-MPA	0.0002
2,4,5-T vs. 4-MPA	<0.0001
2,4,5-T vs. 2,4-DMPA	0.0173
2-MPA vs. 4-MPA	<0.0001
2-MPA vs. 2,4-DMPA	0.7308
4-MPA vs. 2,4-DMPA	<0.0001

Supplementary Table 4: Statistics to support the Binding analyses shown in Fig. 4. A Michaelis–Menten model is fit to describe kinetics.

Substrate	Mutant	r^2	EC_{50} (µM)	I_{max} (nA)	n = x individual sensors
IAA	WT	0.9579	254 ± 6	18 ± 3	3
	S146A	0.8702	498 ± 191	8 ± 2	3
	Y150F	0.8601	452 ± 32	6 ± 2	2
	Y150A	0.9395	1206 ± 298	4 ± 0.3	3
	I51Y	0.8627	547 ± 171	2 ± 0.2	3
	S266A	0.8679	578 ± 183	4 ± 0.6	3
	N223A	0.8533	444 ± 205	2 ± 0.8	3
	S55A	0.8628	213 ± 58	6 ± 1	4
	S55A/S146A	0.7788	341 ± 128	7 ± 2	3
4-CPA	WT	0.9556	800 ± 176	12 ± 2	3
	S146A	0.9073	369 ± 27	8 ± 2	4
	Y150F	0.9167	710 ± 228	5 ± 0.9	4
	Y150A	0.9942	2616 ± 263	5 ± 0.4	4
	I51Y	0.9304	1044 ± 252	3 ± 0.8	3
	S266A	0.8493	2103 ± 290	7 ± 2	3
	N223A	-	-	-	3
	S55A	0.9781	328 ± 15	11 ± 1	3
	S55A/S146A	0.9699	81 ± 3	11 ± 0.9	3
2,4-D	WT	0.9802	179 ± 10	17 ± 2	3
	S146A	0.9476	164 ± 67	8 ± 0.9	4
	Y150F	0.9703	816 ± 275	8 ± 2	3
	Y150A	0.9853	684 ± 256	5 ± 1	4
	I51Y	0.9281	641 ± 77	3 ± 0.8	3
	S266A	0.9800	774 ± 70	12 ± 0.7	3
	N223A	-	-	-	3
	S55A	0.9282	95 ± 36	6 ± 0.6	4
	S55A/S146A	0.8915	38 ± 6	9 ± 2	4
